# Rhinoplasty Combined With Centrofacial Lipofilling to Optimize Facial Proportions

**DOI:** 10.1093/asjof/ojz034

**Published:** 2020-07-11

**Authors:** Marcelo Carvas, Patrick Tonnard, Alexis Verpaele

## Abstract

**Background:**

The perceived appearance of the nose is influenced by its foundations (ie, malar areas, lip, and chin). The association of nasal hump and centrofacial volume deficiency is not uncommon.

**Objectives:**

We evaluated and analyzed the role of centrofacial lipofilling simultaneously to rhinoplasty to sculpt facial proportions and shapes all in one procedure.

**Methods:**

Volumes and placement of fat graft were determined preoperatively. Centrofacial microfat grafting was performed concomitantly to the rhinoplasty. Treated areas were malar, upper lip, pyriform aperture, and chin.

**Results:**

From January 2016 to January 2019, concurrent lipofilling was performed in 23 rhinoplasties. Fat graft volumes ranged from 2 to 31 mL.

**Conclusions:**

Centrofacial lipofilling is a simple and effective tool that can easily be associated with rhinoplasty techniques to optimize the results and may even influence the procedure towards a more conservative approach.

**Level of Evidence: 4:**

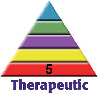

Nasofacial proportions and relationships between facial soft-tissue and bony framework play a role in determining the ideal rhinoplasty for each patient. Preoperative facial and nasal analysis is essential not only to determine surgical steps but also to manage patient’s expectations. Planning the rhinoplasty relies on multiple factors to achieve facial balance that include facial volumes and contours, symmetry, age, sex, and ethnic group.^1-7^

Association of nasal hump and skeletal deficiencies such as malar hypoplasia, periapical hypoplasia, and microgenia is not uncommon. Because of its central position on the face and its relation to surrounding structures, concurrent treatment of the nose and the malar prominence, pyriform aperture, and/or chin may provide optimization of the overall outcome.^8,9^ Creating convexity to a deficient midface alters the perception of an otherwise prominent nose and may influence the surgeon to plan the ideal nose accordingly.^10^ Likewise, in patients with under-projected chin, a nose may appear to project excessively, even though nasal projection may be appropriate to the face.^9,11^

Over the past decade, lipofilling has emerged as an effective and safe alternative to facial implants and/or advancement osteotomies.^12-16^ Autologous fat graft is a durable filler material easily harvested by means of a minimally invasive approach. Advantages of its use as an adjunct to rhinoplasty include low-associated morbidity, ease to precise titration to patient’s specific needs, and long-lasting results. Complications associated with facial lipofilling are considered small and are easily managed. They include oil cysts, lumps, asymmetries, overcorrections, and undercorrections.^12,17^

Despite the remarkable gain in popularity of facial fat grafts and its indications in recent years, few studies have reported the simultaneous association of lipofilling and rhinoplasty. The aim of the present study is to describe and analyze the association of these procedures for a multimodal treatment of facial proportions.

## METHODS

We retrospectively evaluated all concurrent rhinoplasty and centrofacial microfat grafting from January 2016 to January 2019 (consecutive cases) performed by one of the senior authors (P.T.). Preoperative and postoperative photographs were compared. Informed consent was obtained by all patients. According to the principles of the Declaration of Helsinki, all subjects were thoroughly informed of all relevant information regarding both rhinoplasty and lipofilling. Complications and refill procedures (if any) were recorded. Indication for facial lipofilling as an adjunct to the rhinoplasty was provided either after the senior authors’ aesthetic analysis or when the patient actively wanted a change in facial appearance or in its proportions. To date, there is no specific contraindication of lipofilling in our practice. Volumes and placement of fat graft were determined preoperatively and markings of the areas to be augmented were done with the patient in upright position before induction of anesthesia. All procedures were done under general anesthesia and patients were given IV antibiotic (cefazolin 1 g) at the induction of anesthesia.

Microfat harvesting was performed after the infiltration of modified Klein’s solution (1:1.000.000) using a 2.4 mm diameter cannula with 20 sharpened 1-mm holes (Tulip Medical, San Diego, CA). Preferred donor areas were lower abdomen (11 cases, 50%), hips (4 cases, 18.2%), inner thigh (3 cases, 13.6%), lateral thigh (2 cases, 9.1%), anterior thigh (1 case, 4.5%), and gynecomastia (1 case, 4.5%). Preparation of the microfat graft consisted of rinsing the harvested fat with saline over a sterile nylon cloth with 0.5-mm perforations mounted of a sterile cannister. Microfat was then transferred to 1-mL syringes and a blunt 0.7-mm microcannula with a single lateral hole at the end (Tulip Medical, San Diego, CA) was used for grafting.

Microfat grafting was always performed at the beginning of the procedure (before rhinoplasty). This approach minimizes cold ischemia time of the microfat graft and facilitates facial analysis and precise titration of the grafting (once rhinoplasty-related edema is still not present). Also, after the centrofacial volumes are replenished or corrected, rhinoplasty itself can be affected and a more conservative approach may be possible.

Recipients sites were infiltrated with a lidocaine/adrenaline solution (0.3% lidocaine with adrenaline 1:600.000) subcutaneously before initiation of the microfat grafting. After creation of a puncture hole made by a 19-gauge needle, the microcannula was introduced and microfat was deposited through the typical multistroke Coleman technique. For every treated area, 2 access sites are made so that the direction of the tunnels created for grafting is not coincident. Angulation between them varies according to the location. Typically, 90° angle between them is used for malar and tear trough treatment. For upper lip, pyriform, and chin treatment, 1 access site is chosen in each hemiface so that both “grafting tracks” can cross each other with different directions. For malar and tear trough augmentation, fat was grafted at a deep supraperiosteal layer over the maxilla and orbital rim bending into the eyelid. For upper lip, pyriform, and chin treatment, a multilayer approach (from deep and to superficial) was used. Volume of grafting to each area is determined according to surgeon’s experience. For large volumes in 1 location (typically for chin augmentation), intraoperative assessment of tissue’s compliance and expansion also plays a role in determining the amount of grafting. Labiomental crease is also addressed in the same manner to soften it if needed.

## RESULTS

**Figure 1.**
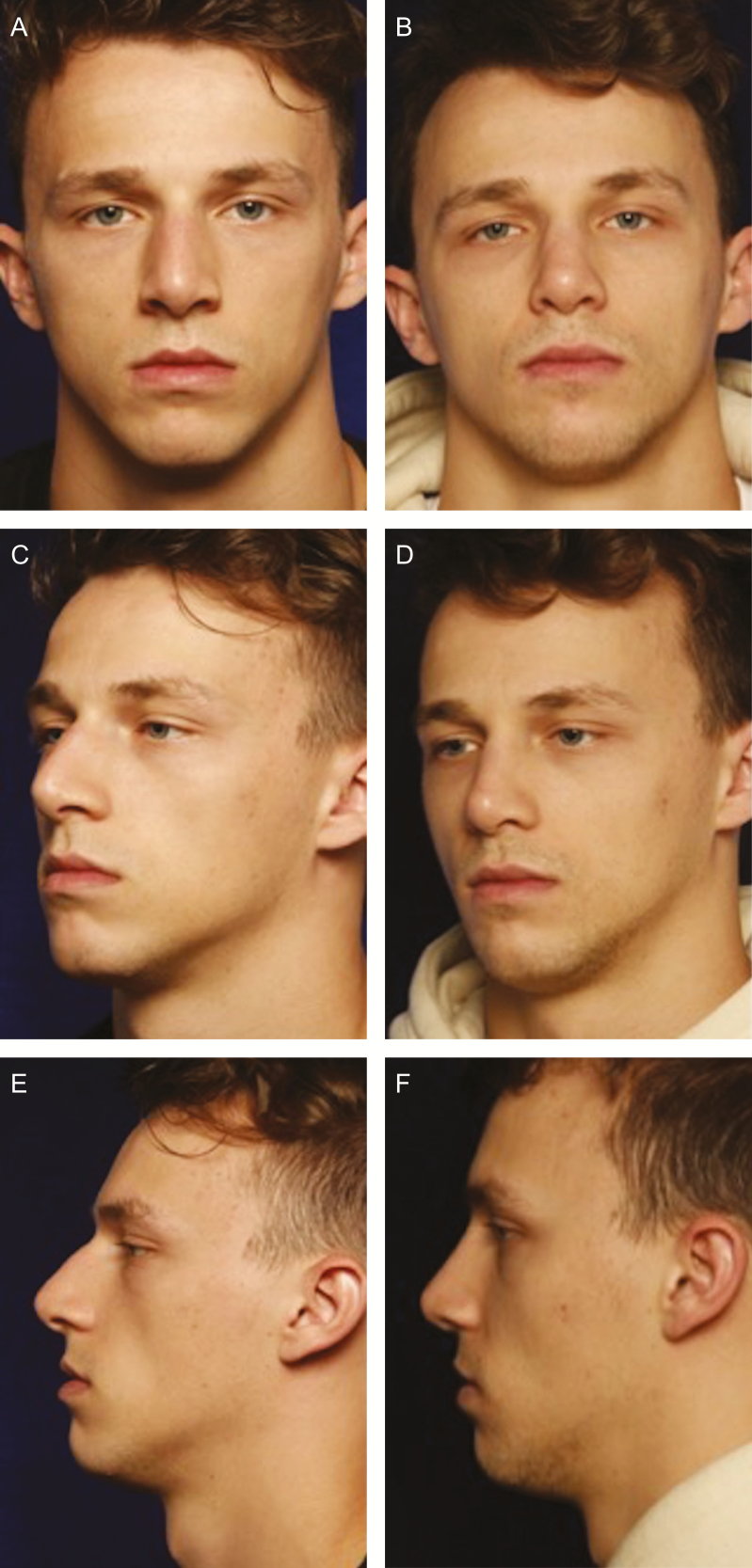
(A, C, E) A 22-year-old male at baseline and (B, D, F) at 7 months follow-up. Rhinoplasty and microfat lipofilling to malar area (5 mL each side). Note the change in the concavity of the midface.

**Figure 2.**
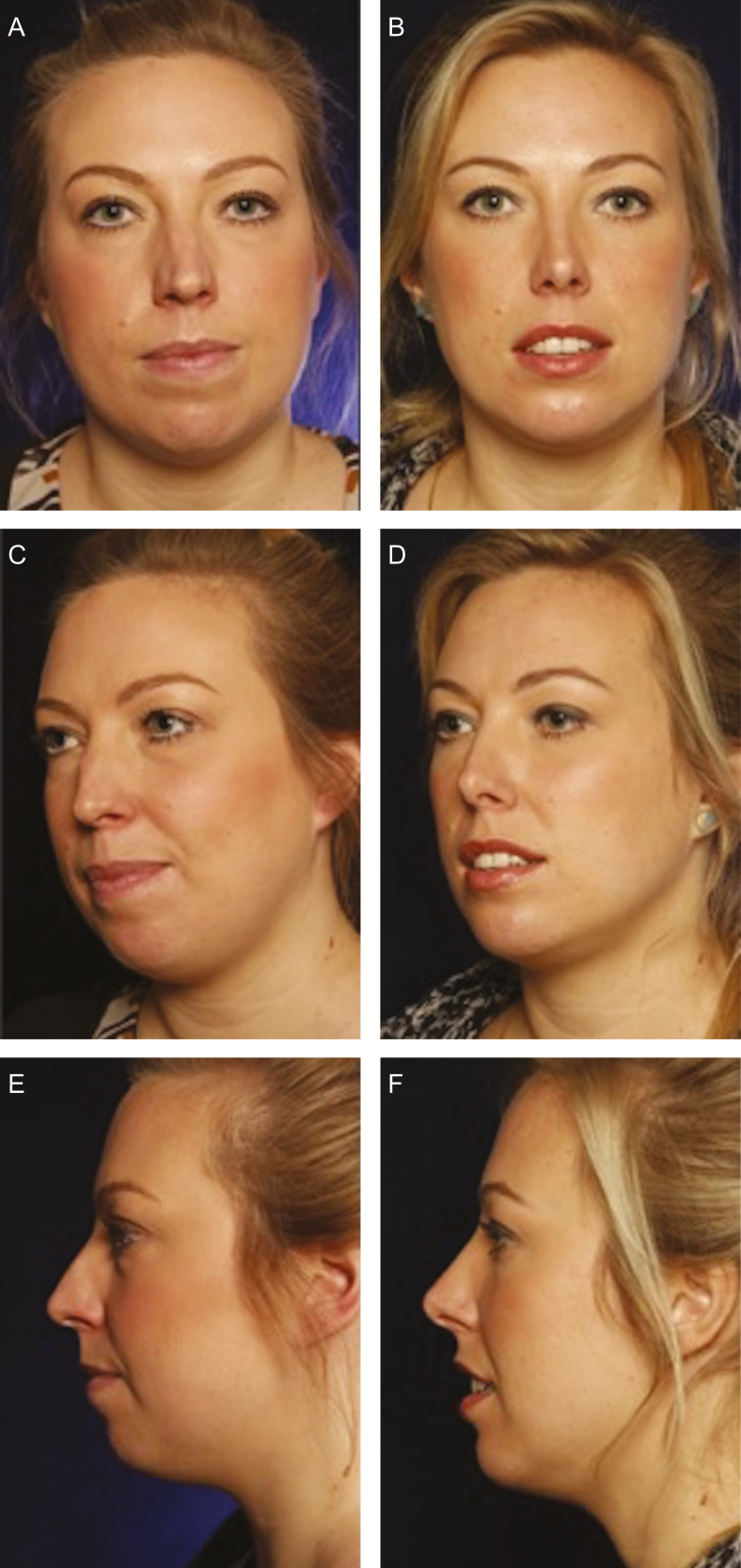
(A, C, E) A 30-year-old female at baseline and (B, D, F) at 12 months follow-up. Rhinoplasty and microfat lipofilling to malar area (4 mL each side) and to chin (14 mL).

From January 2016 to January 2019, 61 rhinoplasties were performed. Concurrent facial fat grafting was associated in 23 of these procedures (37.7%). Among this later group, 15 patients were female (65.2%) and 8 were male (34.8%). The patients’ ages ranged from 22 to 73 years old (mean, 40.9 years). Most commonly recipient sites were malar areas (18 cases, 78.3%), chin (6 cases, 26.1%), and upper lip and pyriform aperture (5 cases, 21.7%). Isolated malar lipofilling was done in 11 cases (47.7%, Figure 1). Asymmetrical malar fat grafting was performed in 4 of these cases (17.42%) to correct malar asymmetry. In 5 patients (21.7%), both malar area and chin were addressed concurrently in adjunct to the rhinoplasty (Figure 2). No microfat grafting was done to the nose or to correct nasal irregularities in any patient.

Injected volumes of microfat ranged from 2 to 11 mL (median, 5 mL) per side per malar area, 8 to 20 mL (median, 14 mL) to the chin, and 2 to 31 mL (median, 7 mL) to upper lip. Follow-up ranged from 1 to 29 months (median, 4.5 months) and during that period no complications were recorded nor was any refill procedure done.

## DISCUSSION

Symmetry and balanced facial proportions are considered key components not only for the impression of beauty but also of youthfulness. Many systematic evaluations have been proposed for a full-face analysis.^10,18-20^ Due to the central position of the nose, the surrounding facial structures play a role in determining the visual impression of the nose itself.^8,9,11,12^ Since the nasal pyramid sits between the malar areas, a deficient midface may alter the perceived appearance of the nose and may render it larger than it really does. This may be due to a congenital or acquired bony deficiency but also to the deflation due to the aging process.^21-23^

**Figure 3.**
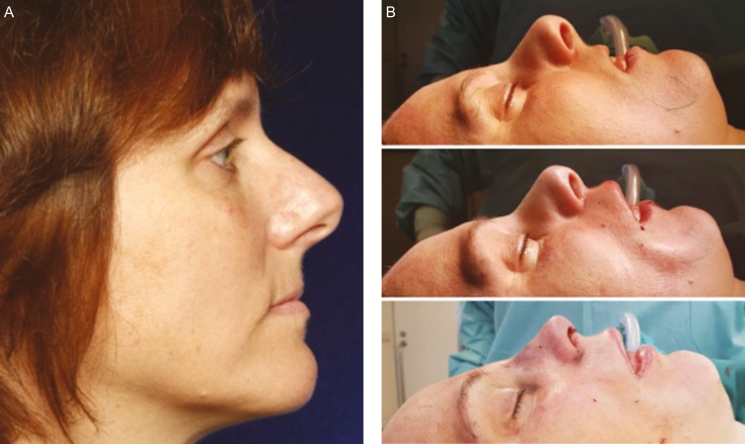
The influence of lipofilling to the upper lip on the perceived appearance of the nose demonstrated on a 48-year-old female. (A) Preoperative profile photograph shows low projection of the upper lip associated with an over-projected nose. (B, top) The intraoperative supine profile view shows baseline relation between upper lip and nose before any work is done. (B, middle) Lipofilling alone done to the upper lip (10 mL) influences of the perceived projection of the nose before initiation of rhinoplasty. (B, bottom) Immediate postoperative view shows the effect of combining rhinoplasty to lipofilling.

Similarly, an under-projected upper lip may create the impression of an overly projected nose (Figure 3). The underlying causes include retruded maxilla, periapical hypoplasia, and aging process. Ramaut et al.^24^ recently reported the age-related changes in the upper lip such as loss of volume, lengthening, and thinning based on MRI measurements in young compared with an in elderly population. With regard to the skeletal aging, pyriform aperture widening^8,22,23^ may also play a role in changing the projection of the upper lip.

**Figure 4.**
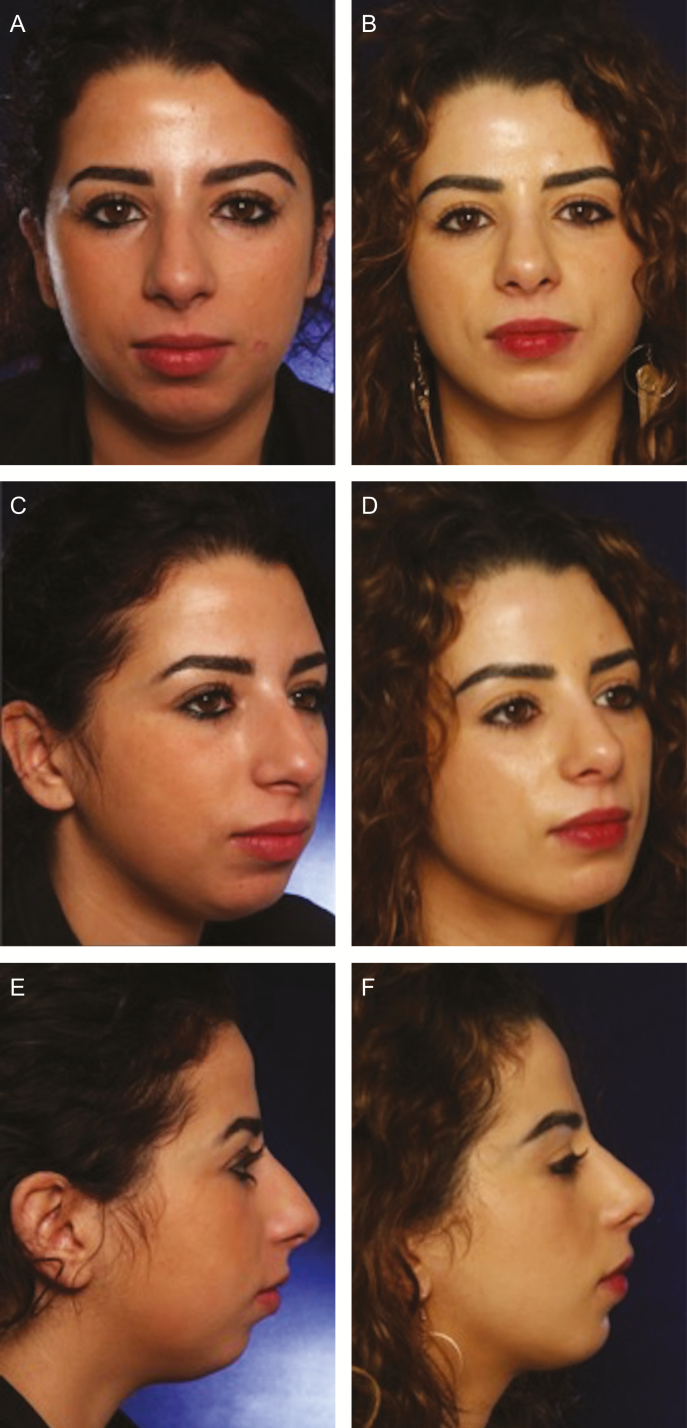
(A, C, E) A 24-year-old female at baseline and (B, D, F) at 27 months follow-up. Profiloplasty: rhinoplasty and microfat lipofilling exclusively to chin (20 cc). Long-term follow-up shows persistent result.

The neck and the chin also influence the perceived appearance of the nose. Using computer-altered photographs of necks, Greer et al.^11^ found that nonaltered noses were rated to have a “better” appearance in better-contoured necks after image editing. Likewise, in patients with under-projected chins, the nose appears to project excessively, even though nasal projection may be appropriate to the face.^9,12^ This is particularly true when the nasofacial proportions are analyzed in the profile view. Addressing nose and chin simultaneously in a so-called profiloplasty may optimize results (Figure 4).

Historically, a multitude of alloplastic implants was described to provide a more youthful and proportionated appearance, treating congenital or acquired skeletal deficiency and/or correcting facial asymmetry.^8,25-31^ However, short- and long-term complications of facial implants include displacements/malposition, prominence problems, bony resorption, transient or permanent nerve injury, infections, capsule formation, and scaring.^32-35^

**Figure 5.**
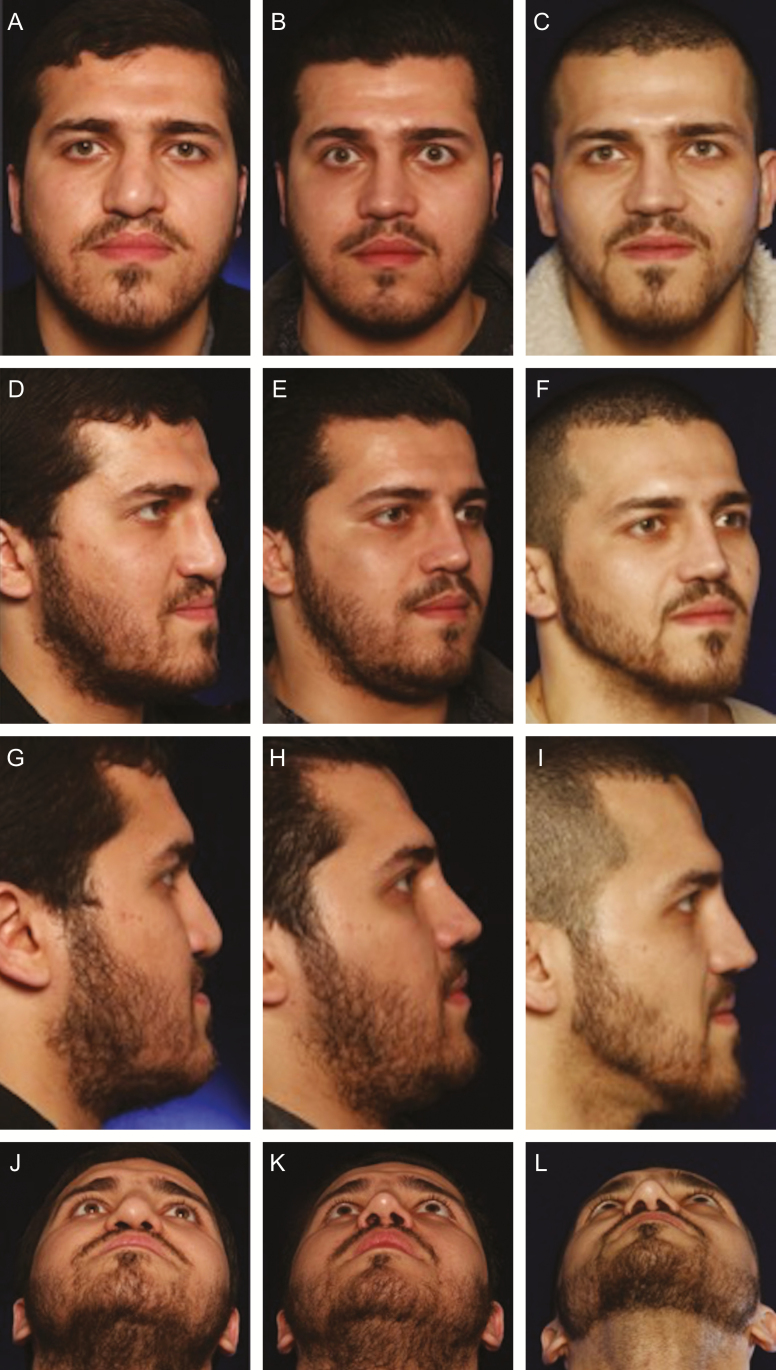
(A, D, G, J) This 25-year-old male previously underwent 3 rhinoplasties elsewhere. Quaternary rhinoplasty was done using rib graft and 31 mL of microfat graft for treatment of periapical hypoplasia and upper lip retrusion. (B, E, H, K) At 4 months follow-up, note the change in the previously concave and under-projected midface. (C, F, I, L) At 29 months follow-up, even though patient had lost 40 kg, a durable result of the lipofilling is seen.

Initially used as an adjunct to facelifts and facial rejuvenation procedures to replenish age-related volume loss,^14-16,36^ lipofilling has gained a wider spectrum of indications with its ability to also achieve additional projection. Its use was associated as an alternative to facial implants and advancements osteotomies.^12^ To date, the authors consider microfat grafting as effective in replenishing age-related volume loss as it is for correction of skeletal deficiency. One of the patients in the present study had 3 previous unsuccessful rhinoplasties and presented to us with an under-projected nose corrected with rib cartilage graft and with a severe midface retrusion and periapical hypoplasia corrected by 31 mL of facial fat injection to the upper lip and pyriform aperture (Figure 5).

**Figure 6.**
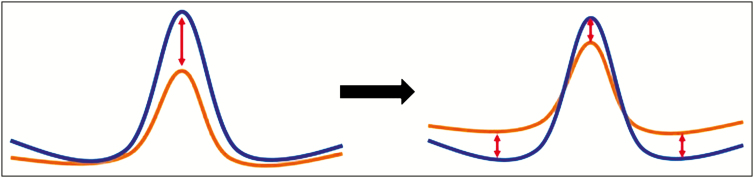
Combination of lipofilling and rhinoplasty may shift to procedure to a more conservative approach.

With the recent trend toward more conservative techniques in rhinoplasty,^37^ correcting centrofacial volumes and shapes as an adjunct to rhinoplasty may affect the classical reductional rhinoplasty. A commonly used comparison is a mountain surrounded by its valleys. Instead of exclusive reduction of the mountain, a combined filling of the valleys reduces the extend of resection and reshaping of the relief (Figure 6).

Advantages of this concomitant lipofilling include the minimal lengthening of surgical procedure, low-associated morbidity, the ease to precise titration of patient’s needs, and the possibility to correct asymmetries. Although resorption rates vary, a refill procedure can easily be performed under local anesthesia if needed. Our previous study suggested the resorption ranges from 15% in the immobile malar and chin areas to 50% in the mobile lip and chin area.^12^

Limitations of our study are its retrospective character, the limited sample size, and the short-term follow-up limiting the global estimation of fat graft resorption and the need of refill procedures. Also, patient’s perceived outcomes were not evaluated.

## CONCLUSIONS

Centrofacial lipofilling is a simple and effective tool that can easily be associated with rhinoplasty techniques to optimize the results and may even influence the rhinoplasty towards a more conservative approach.
